# Comparing Multi-Criteria Analysis and Species Distribution Models for Identifying Locust Suitable Habitats in Xinjiang, China

**DOI:** 10.3390/biology15100736

**Published:** 2026-05-07

**Authors:** Sijie Cui, Jianghua Zheng, Jun Lin, Zhong Liang, Feifei Zhang, Junteng Luo, Xuan Li, Xiaoyu Guo, Jianguo Wu

**Affiliations:** 1 College of Geography and Remote Sensing Science, Xinjiang University, Urumqi 830046, China; 107552303751@stu.xju.edu.cn (S.C.); zhangfeifei@stu.xju.edu.cn (F.Z.); 107556524203@stu.xju.edu.cn (J.L.); 107556523284@stu.xju.edu.cn (X.L.); 107552301174@stu.xju.edu.cn (X.G.); 2 Xinjiang Key Laboratory of Oasis Ecology, Urumqi 830046, China; 3 Center for Grassland Biological Disaster Prevention and Control of Xinjiang, Urumqi 830001, China; xjcy2009@163.com (J.L.); 18999280970@163.com (Z.L.); zhbshuju@163.com (J.W.)

**Keywords:** multi-criteria analysis, species distribution models, locust suitable habitat, spatial agreement

## Abstract

Locust outbreaks are major biological disturbances in grassland ecosystems of arid and semi-arid regions. Accurate identification of locust suitable habitats is important for regional monitoring and management. However, direct comparisons between multi-criteria analysis (MCA) and species distribution models (SDMs) under a unified framework remain limited. In this study, we compared these two approaches for dominant locust species in Xinjiang, China, including *Calliptamus italicus*, *Gomphocerus sibiricus*, and *Locusta migratoria manilensis.* We used the same environmental variables and occurrence records for all models. The MCA methods included the analytic hierarchy process (AHP), technique for order preference by similarity to ideal solution (TOPSIS), and ordered weighted averaging (OWA). The SDMs included the generalized linear model (GLM), maximum entropy model (MaxEnt), extreme gradient boosting (XGBoost), and an ensemble model. The results showed that SDMs had higher area under the receiver operating characteristic curve (AUC) and true skill statistic (TSS) values than MCA under the internal point-based evaluation framework, although both approaches effectively identified locust-suitable habitats. The two approaches also showed high spatial agreement in moderately and highly suitable habitats, with Jaccard indices of 0.88–0.92, and consistently identified the northern slopes of the Tianshan Mountains, the Ili River Valley, and the margins of the Junggar Basin as core suitable areas. These results indicate that the two approaches are complementary for locust monitoring and management.

## Simple Summary

Locust outbreaks are a major threat to grasslands in arid regions and may affect ecological stability. This study compared two commonly used mapping methods. One approach was based on ecological knowledge and environmental conditions, while the other was based on species occurrence records and statistical prediction. Using the same environmental data and locust records, we found that both methods could identify potential risk areas. However, the data-driven methods showed stronger point-based discrimination and identified more concentrated high-risk areas. Both approaches consistently identified the northern slopes of the Tianshan Mountains, the Ili River Valley, and the margins of the Junggar Basin as key suitable areas. For practical use, the expert-based method can support broad monitoring and early warning, while the data-driven method can refine priority hotspots for field surveys and targeted control. Their combined use can improve decision-making and the allocation of monitoring and control resources for grassland pest management.

## 1. Introduction

Locust outbreaks are important biological disturbances in arid and semi-arid grassland ecosystems. By consuming aboveground biomass and altering vegetation structure, locusts can reduce forage availability, affect grassland productivity, and modify ecosystem carbon exchange [1,2]. These effects are not only ecological, but also agro-productive and economic, because grassland productivity is closely related to forage supply, livestock production, pest control costs, and the allocation of monitoring resources in pastoral regions. Accurate identification of locust suitable habitats is therefore important for regional monitoring, risk assessment, and locust management [3].

Remote sensing and geographic information systems have improved the availability of climate, vegetation, soil, hydrological, and topographic data. These data are now widely used for locust habitat monitoring, suitability mapping, and risk assessment across multiple spatial scales [4,5,6,7]. Two broad modeling approaches are commonly used for this purpose. The first is multi-criteria analysis (MCA), which evaluates environmental suitability through ecological knowledge, indicator grading, and weight assignment [8,9,10,11,12,13,14]. The second is species distribution models (SDMs), which estimate potential distributions by relating occurrence records to environmental variables [15,16,17,18,19]. These two approaches differ in both data requirements and sources of uncertainty. MCA can incorporate ecological knowledge and environmental constraints when occurrence records are limited or unevenly distributed. However, outputs from MCA may vary with indicator classification, weighting schemes, and decision strategies [9,10,12]. SDMs often provide strong predictive performance, but they are sensitive to sampling bias, background selection, and pseudo-absence settings [15,18,19]. Ensemble forecasting and multi-model platforms are therefore often used to reduce model uncertainty and improve robustness [17,20,21].

MCA and SDMs are therefore not fully equivalent modeling approaches. MCA is a knowledge-driven and decision-support framework. It uses ecological knowledge, suitability grading, and weighting rules to represent potential environmental suitability. In contrast, SDMs are occurrence-driven correlative models. They estimate statistical relationships between occurrence records and environmental variables and are more closely related to realized occurrence patterns. This distinction is important because evaluation metrics such as evaluation metrics such as the area under the receiver operating characteristic curve (AUC) and true skill statistic (TSS) are designed to measure discrimination between occurrence and absence or pseudo-absence samples. These metrics may therefore favor occurrence-driven models and should not be used alone to judge the overall ecological validity or management value of MCA and SDMs.

Previous studies have applied MCA, SDMs, and related geospatial approaches to locust habitat assessment, breeding area identification, and future habitat prediction under climate change scenarios [20,22,23,24,25,26,27]. However, most studies have focused on a single modeling framework or on context-specific applications. Direct comparisons between knowledge-driven MCA and occurrence-driven SDMs under the same environmental variables and occurrence records remain limited. This gap is important because different modeling logics may lead to different suitability maps, different interpretations of high-risk areas, and different implications for monitoring and control. A unified comparison can therefore help clarify not only which approach has stronger point-based discrimination, but also how the two approaches differ in spatial delineation and management use.

Against this background, this study selected dominant locust species in Xinjiang, represented by *Calliptamus italicus*, *Gomphocerus sibiricus*, and *Locusta migratoria manilensis*, and compared MCA and SDMs under a unified environmental variable system and occurrence dataset. Specifically, the analytic hierarchy process (AHP), the technique for order preference by similarity to ideal solution (TOPSIS), ordered weighted averaging (OWA) were compared with the generalized linear model (GLM), maximum entropy model (MaxEnt), extreme gradient boosting (XGBoost), and an ensemble model. Based on the different modeling logics of the two approaches, we tested three methodological expectations. First, SDMs were expected to show higher point-based discrimination than MCA because they are calibrated directly with occurrence records. Second, MCA and SDMs were expected to show broad spatial agreement in moderately and highly suitable habitats because both approaches use environmental gradients related to locust occurrence. Third, the two approaches were expected to differ in the delineation of highly suitable habitats because MCA represents potential environmental suitability, whereas SDMs are more strongly constrained by realized occurrence records. The aim was to clarify the relative strengths and limitations of the two approaches and to provide methodological support for locust habitat assessment, early warning, and pest risk management in arid and semi-arid grassland regions.

## 2. Materials and Methods

### 2.1. Study Area and Occurrence Records

**Figure 1:**
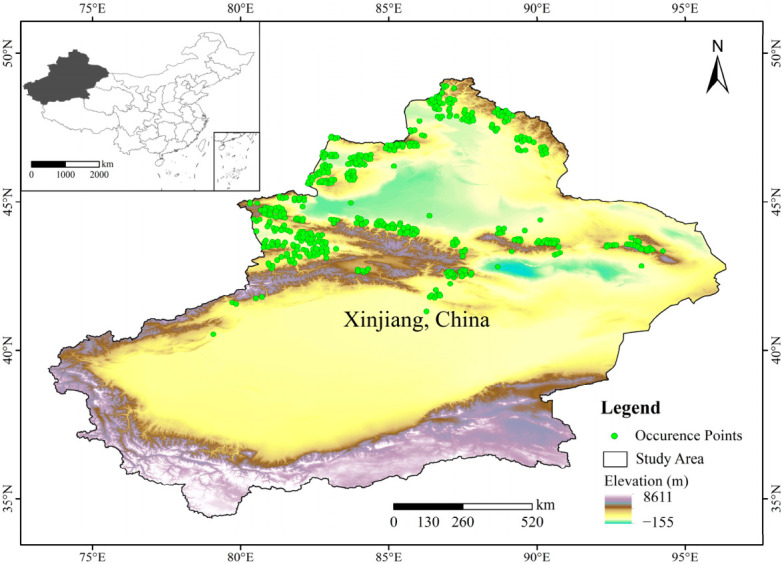
Study area and locust occurrence records.

Xinjiang is situated in Northwestern China, between 73°40′ E and 96°18′ E, and 34°25′ N and 49°10′ N. The region as a whole exhibits a topographical pattern characterized by ‘three mountain ranges flanking two basins’, with a distinctly undulating terrain and significant variations in altitude. The region has a typical temperate continental arid climate, with scarce precipitation that is unevenly distributed in both time and space and has marked variations in temperature. This study focused on three dominant locust species, namely *Calliptamus italicus*, *Gomphocerus sibiricus*, and *Locusta migratoria manilensis* [28]. Because the objective of this study was to compare MCA and SDMs for regional-scale locust habitat assessment, these species were combined to represent the composite suitability pattern of dominant grassland locusts in Xinjiang, China rather than the ecological niche of a single species. Occurrence records of these species were compiled from field surveys conducted by the research team and from previously published literature [29,30]. A total of 1618 records of *Calliptamus italicus*, 1317 records of *Gomphocerus sibiricus*, and 36 records of *Locusta migratoria manilensis* were initially collected. To reduce spatial autocorrelation and sampling bias, duplicate records were removed, and the Spatially Sparse Occurrence Data tool in SDMtoolbox (version 2.6) was used to perform spatial thinning with a minimum distance of 5 km between occurrence points [31]. After filtering, 480 occurrence records were retained for subsequent analyses (Figure 1).

### 2.2. Environmental Variables

Environmental variables were selected from five categories, including climate, vegetation, soil, hydrology, and topography. Climate variables included land surface temperature (LST) [32], air temperature [33,34], precipitation [35], and soil moisture [36], which were used to characterize the thermal and moisture conditions associated with locust occurrence. Vegetation variables included normalized difference vegetation index (NDVI) [37] and land cover type [38], which were used to represent vegetation growth conditions and habitat background. Soil variables included clay content, sand content, and pH at a depth of 5–15 cm, reflecting substrate conditions related to locust oviposition and development [39]. Hydrological information on rivers and lakes was used to represent water related environmental constraints [40], and SRTM DEM was used to characterize topographic conditions [41]. Detailed information on the data sources, temporal resolution, and spatial resolution of each variable is provided in Table 1.

All environmental datasets were cropped to the Xinjiang region of China, projected into the World Geodetic System 1984 coordinate system coordinate system, and standardized to a spatial resolution of 1 km. Climate and vegetation variables from 2001 to 2022 were aggregated into multi-year average conditions for the locust development period (May–August) and the overwintering period (November–February of the following year). The May–August period was used to represent key thermal, moisture, and vegetation conditions during locust development, whereas the November–February period was used to represent overwintering related environmental constraints. This treatment was used to characterize long-term environmental suitability rather than year-specific outbreak risk. The DEM was used to calculate slope and aspect, while hydrological variables were converted into layers representing Euclidean distances to the nearest rivers and lakes. To reduce multicollinearity, candidate variables were screened using Spearman’s correlation analysis and the MaxEnt jackknife test. Where the absolute correlation coefficient exceeded 0.75, variables with clearer ecological significance and a stronger association with locust occurrence were retained. Ultimately, 12 variables were selected for subsequent MCA and SDMs modeling. To support MCA modeling, the selected environmental variables were converted into five suitability scores according to published studies, locust ecological preferences, and the environmental characteristics of Xinjiang, China [14,27,42,43,44]. The suitability scores were used to represent the relative favorability of different environmental conditions for locust occurrence. For each variable, higher scores indicated conditions considered more favorable for locust occurrence based on previous studies and regional ecological knowledge. The grading criteria are shown in Table 2.

### 2.3. Multi-Criteria Analysis

Three multi-criteria analysis methods were used in this study, namely the analytic hierarchy process (AHP), the technique for order preference by similarity to ideal solution (TOPSIS), and the ordered weighted averaging (OWA). These methods were selected because they represent different decision logics for integrating multiple environmental variables into locust habitat suitability assessment. The standardized suitability scores of the selected environmental variables were used as common inputs for all MCA models.

#### 2.3.1. Analytic Hierarchy Process (AHP)

In the AHP model, the relative importance of environmental variables was determined through pairwise comparison using the 1 to 9 scale method [45]. The pairwise comparisons were based on published studies, regional ecological knowledge of dominant grassland locusts in Xinjiang, China, and the ecological meaning of each environmental variable. Judgment matrices were constructed within each hierarchical level, and consistency was evaluated using the consistency ratio. Matrices with a consistency ratio lower than 0.1 were considered acceptable. After weight determination, the habitat suitability index for each grid cell was calculated by weighted linear combination:

(1) $$HSI_{i}=\sum_{j=1}^{n}w_{j}x_{ij}$$

where *HSI_i_* is the habitat suitability index of grid cell *i*, *w_j_* is the weight of variable *j*, and *x_ij_* is the standardized suitability score of variable *j* in grid cell *i*. Larger values indicate higher habitat suitability for locust occurrence.

#### 2.3.2. Technique for Order Preference by Similarity to Ideal Solution (TOPSIS)

TOPSIS was used to rank the suitability of each grid cell according to its relative proximity to the positive ideal solution and its distance from the negative ideal solution in a weighted multi-variable space [46]. First, a weighted standardized decision matrix was constructed:

(2) $$v_{ij}=w_{j}\cdot r_{ij}$$

where *v_ij_* is the weighted standardized value of variable *j* in grid cell *i*, *w_j_* is the weight of variable *j*, and *r_ij_* is the standardized suitability score.

The positive ideal solution *A*^+^ and negative ideal solution *A*^−^ were then defined as:

(3) $$A^{+}=\left\{\max(v_{ij})|j=1,2,\ldots ,n\right\}$$

(4) $$A^{-}=\left\{\min(v_{ij})|j=1,2,\ldots ,n\right\}$$

The Euclidean distances from each grid cell to the positive and negative ideal solutions were calculated as:

(5) $$D_{i}^{+}=\sqrt{\sum_{j=1}^{n}{\left(v_{ij}-A_{j}^{+}\right)}^{2}}$$

(6) $$D_{i}^{-}=\sqrt{\sum_{j=1}^{n}{\left(v_{ij}-A_{j}^{-}\right)}^{2}}$$

Finally, the relative closeness of each grid cell to the ideal solution was calculated as:

(7) $$C_{i}=\frac{D_{i}^{-}}{D_{i}^{+}+D_{i}^{-}}$$

where *C_i_* ranges from 0 to 1, and larger values indicate higher locust habitat suitability.

#### 2.3.3. Ordered Weighted Averaging (OWA)

OWA is a multi-criteria decision method that represents different decision attitudes by assigning order weights to the ranked suitability values of environmental variables within each grid cell [47]. This method allows continuous transitions among optimistic, neutral, and conservative decision strategies, and is therefore suitable for habitat suitability assessment under uncertainty [48]. For grid cell *i*, the standardized suitability values of the n environmental variables were first arranged in descending order as *z_i_*_1_ ≥ *z_i_*_2_ ≥ ⋯ ≥ *z_in_*. The OWA suitability index was then calculated as:

(8) $$OWA_{i}=\sum_{j=1}^{n}u_{j}z_{ij}$$

where *OWA_i_* is the suitability index of grid cell *i*, *z_ij_* is the *j*-th ranked standardized suitability value, and *u_j_* is the order weight assigned to rank *j*, with $\sum_{j=1}^{n}u_{j}=1$.

The order weights were generated using the decision parameter α as follows:

(9) $$u_{j}={\left(\sum_{k=1}^{j}r_{k}\right)}^{\alpha }-{\left(\sum_{k=1}^{j-1}r_{k}\right)}^{\alpha }$$

where *r_k_* is the reordered criterion weight corresponding to the ranked suitability value, obtained by rearranging the original variable weights according to the descending order of *z_ij_*, and $\sum_{k=1}^{n}r_{k}=1$. When *α* < 1, larger weights are assigned to higher ranked values, representing a more optimistic decision strategy. When *α* = 1, the model approximates a neutral weighted average. When *α* > 1, greater weights are assigned to lower ranked values, representing a more conservative decision strategy.

### 2.4. Construction and Evaluation of Species Distribution Models

Four species distribution models, namely GLM, MaxEnt, XGBoost, and an ensemble model, were used in this study. These models were implemented using the biomod2 package (version 4.3-4-3) in R software (version 4.4.3). They were selected to represent different modeling strategies, including parametric statistical modeling, presence background modeling, machine learning, and multi-model integration.

Model construction was based on the occurrence records described above and pseudo-absence points generated within the biomod2 framework. Pseudo-absence points were randomly generated from the study area at a presence-to-pseudo-absence ratio of 1:3. To avoid overlap between presence and pseudo-absence samples, pseudo-absence points located in the same raster cells as occurrence records were removed. The same set of pseudo-absence points was used for all SDMs to maintain comparability among model outputs [49]. All models were developed using the same occurrence dataset and the same set of environmental variables to ensure comparability among model outputs. For MaxEnt, model calibration was optimized using the ENMeval package (version 2.0.5.2) [50]. The final parameter settings were a regularization multiplier of 3 and a linear feature class. For GLM and XGBoost, the standard settings in the biomod2 framework were used to maintain a consistent modeling framework across algorithms. This setting was intended to support methodological comparison under unified inputs rather than to fully optimize each algorithm independently. Model performance was evaluated using 10-fold cross validation, and each model was repeated 10 times to reduce the influence of random partitioning. Predictive accuracy was assessed using the area under the receiver operating characteristic curve (AUC) and the true skill statistic (TSS). An AUC value greater than 0.8 was considered to indicate good or very good predictive performance [51]. TSS ranges from 0 to 1 and reflects the net predictive success of the model for the evaluation samples. A TSS value greater than 0.7 was considered to indicate high predictive accuracy [52]. Based on the evaluation results, model outputs with TSS values greater than 0.7 and AUC values greater than 0.9 were selected for ensemble construction. In the ensemble procedure, each individual model was assigned a weight according to its TSS score, so that models with higher predictive accuracy contributed more strongly to the final suitability prediction.

### 2.5. Habitat Suitability Classification

To satisfy both spatial mapping and quantitative model comparison, two complementary suitability classification strategies were used in this study. For spatial mapping, the raw outputs of each model were first linearly normalized to the range of 0 to 1. The suitable habitats were then classified using a combination of the Jenks natural breaks method and empirical thresholds into unsuitable habitats (0 to the first natural break), slightly suitable habitats (from the first natural break to 0.6), moderately suitable habitats (0.6 to 0.8), and highly suitable habitats (0.8 to 1.0) [27,53,54]. This classification scheme was used to improve the clarity and ecological interpretability of the spatial distribution maps.

Because MCA and SDMs differ in model structure and output characteristics, the linearly normalized values were not directly comparable across models. Therefore, cumulative distribution function (CDF) normalization was applied in the model comparison stage [55]. By converting each grid cell value into its percentile within the regional distribution, CDF normalization transformed different model outputs into a comparable 0 to 1 probability scale and reduced differences in value range and distribution shape among models [31]. The CDF normalized outputs were then classified using the same four-level structure for quantitative comparison.

## 3. Results

### 3.1. Model Evaluation

AUC and TSS were used to evaluate the internal point-based discrimination ability of each model based on occurrence and pseudo-absence samples (Table 3). These metrics indicate how well model outputs separated occurrence records from pseudo-absence points, rather than the overall ecological validity or management value of each modeling approach. Because MCA produces deterministic suitability surfaces, whereas SDMs were evaluated through repeated cross-validation, formal significance testing between MCA and SDM evaluation values was not conducted. Therefore, the AUC and TSS results are interpreted descriptively and are complemented by occurrence hit rates, CDF-normalized area comparisons, and Jaccard spatial overlap analysis.

Except for OWA with α = 0.0001 and OWA with α = 10,000, all models achieved AUC values above 0.90, indicating strong discrimination between presence and pseudo-absence samples under the selected evaluation framework. Among the MCA models, AUC values ranged from 0.632 to 0.943. AHP and TOPSIS showed similar performance, whereas the point-based discrimination performance of OWA varied substantially with the weight parameter α. In comparison, SDMs generally showed higher AUC values than MCA under the occurrence and pseudo-absence evaluation framework. The ensemble model achieved the highest AUC value (0.982), indicating strong point-based discrimination by the ensemble model.

The TSS results further indicated clear differences among models in threshold-based evaluation. Excluding OWA with α = 0.0001 and OWA with α = 10,000, the MCA models produced TSS values ranging from 0.659 to 0.726. In contrast, the TSS values of the SDMs were consistently higher, ranging from 0.836 to 0.851. Among them, XGBoost achieved the highest TSS value (0.851), indicating the strongest threshold-based discrimination among the evaluated models. Taken together, the AUC and TSS results indicated that SDMs had stronger point-based discrimination ability than MCA under the selected internal evaluation framework.

### 3.2. Spatial Patterns of Locust Suitable Habitats Based on MCA

**Figure 2:**
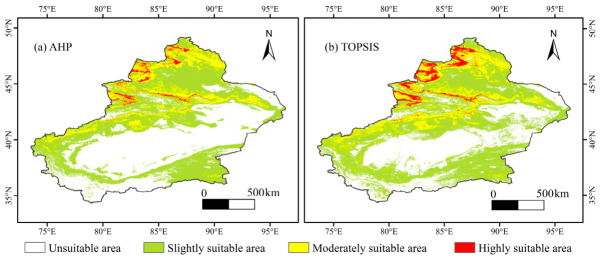
Habitat suitability maps for the locust based on (**a**) AHP and (**b**) TOPSIS.

**Figure 3:**
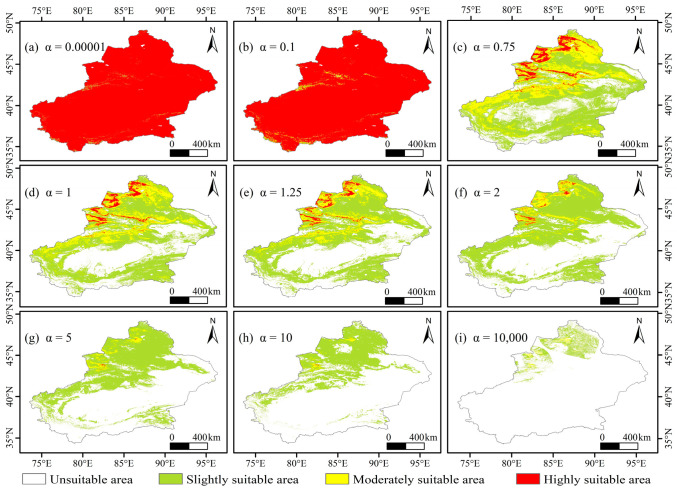
Habitat suitability maps for the locust based on OWA under different α.

Based on the AHP derived attribute weights (Table A1), habitat suitability maps generated by the MCA framework are shown in Figure 2 and Figure 3. The AHP and TOPSIS results exhibited broadly similar spatial patterns. In both models, highly suitable habitats were mainly concentrated on the southern slopes of the Altai Mountains, the central northern slopes of the Tianshan Mountains, the Ili River Valley, and the Western Tacheng Emin Basin region, where they formed relatively continuous belt-like and patchy distributions. Compared with AHP, TOPSIS identified a smaller extent of highly suitable habitats. Moderately suitable habitats were mainly distributed around the highly suitable habitats and formed transitional zones. Slightly suitable habitats were mainly distributed in central and Southern Xinjiang, China, especially in the central and Southern Junggar Basin, the margins of the Tarim Basin, and the areas south of the Tianshan Mountains. Unsuitable habitats in both models were mainly concentrated in the interior of the Tarim Basin, the Kunlun Mountains, high-elevation areas of the Tianshan Mountains, and the desert areas of Southern Xinjiang, China. The area proportions derived from MCA further showed differences among methods. Under AHP, unsuitable, slightly suitable, moderately suitable, and highly suitable habitats accounted for 35.44%, 46.49%, 14.88%, and 3.18%, respectively (Table A2). Under TOPSIS, the corresponding proportions were 44.80%, 41.20%, 12.41%, and 1.59%, indicating that TOPSIS produced a more restricted highly suitable habitat pattern than AHP.

The OWA results revealed strong sensitivity to the order weight parameter α. The scenarios with α = 0.0001, α = 0.1, and α = 10,000 were interpreted as boundary cases representing extremely optimistic or extremely conservative decision attitudes, rather than as realistic management scenarios. When α = 0.0001 and 0.1, highly suitable habitats accounted for 98.71% and 97.47% of the study area, respectively, whereas the combined proportion of moderately and slightly suitable habitats remained below 2%, and unsuitable habitats were almost absent. These results indicate that extremely optimistic decision attitudes may overexpand suitable habitats and reduce the practical interpretability of the output. As α increased to 0.75–1.25, the extent of highly suitable habitats decreased markedly, and the suitability structure became more stable, with moderately and highly suitable habitats gradually concentrating in typical areas such as the northern slopes of the Tianshan Mountains and the Ili River Valley. When α = 1, the OWA output approximated a weighted average and was therefore highly consistent with the AHP result. As α increased further, highly suitable habitats continued to shrink and became scattered only in parts of Northern Xinjiang, China, while moderately suitable habitats occurred as small patches around them. Under extremely large α values, only limited slightly and moderately suitable habitats remained, and most of the study area was classified as unsuitable. This suggests that extremely conservative decision attitudes may underrepresent potential suitable habitats. Based on the model evaluation results and spatial interpretability, the OWA scenario with α = 2 was retained as the representative MCA result for subsequent comparison with SDMs, reflecting habitat delineation under a relatively conservative decision attitude. Under this scenario, unsuitable, slightly suitable, moderately suitable, and highly suitable habitats accounted for 45.79%, 47.82%, 5.69%, and 0.70%, respectively.

### 3.3. Spatial Patterns of Locust Suitable Habitats Based on SDMs

**Figure 4:**
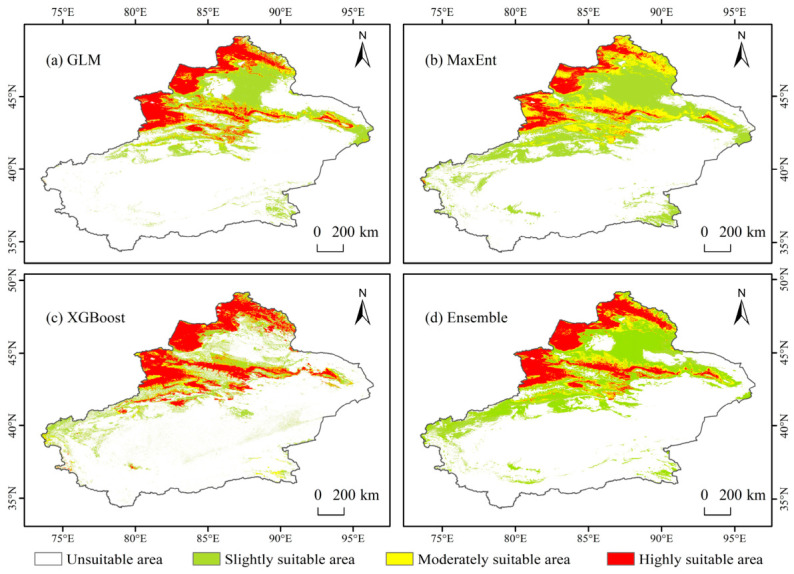
Habitat suitability maps for the locust based on SDMs.

The four SDMs showed broadly consistent spatial patterns of locust suitable habitats in Xinjiang, China, with the main suitable areas concentrated on the southern slopes of the Altai Mountains, the central northern slopes of the Tianshan Mountains, the Ili River Valley, and the margins of the Junggar Basin (Figure 4). Highly suitable habitats were mainly distributed as continuous belts along the piedmont areas and river valleys of Northern Xinjiang, China. Moderately suitable habitats were generally located around the highly suitable habitats, whereas slightly suitable habitats were scattered within the interior basin areas and parts of Southern Xinjiang, China. In contrast, unsuitable habitats were mainly concentrated in the interior of the Tarim Basin and in high-elevation mountainous areas.

Clear differences were observed among the four models in the spatial extent and area structure of highly suitable habitats (Table A3). Among them, XGBoost predicted the largest proportion of highly suitable habitats (16.18%), whereas MaxEnt produced the most concentrated pattern, with highly suitable habitats accounting for only 7.29% of the study area. The GLM result was intermediate (15.02%), while the ensemble model produced a relatively convergent prediction, with highly suitable habitats accounting for 9.64%. Moderately suitable habitats occupied relatively small proportions in all four models.

### 3.4. Comparison of Suitability Patterns After CDF Normalization

**Figure 5:**
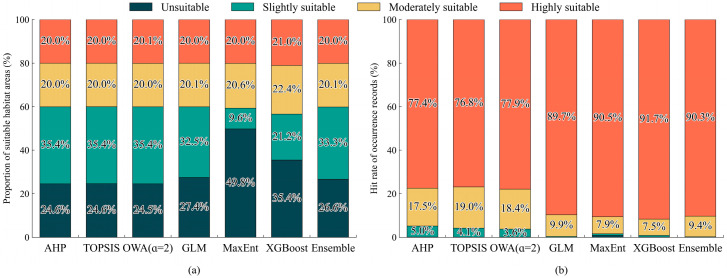
(**a**) Proportion of suitable habitat areas and (**b**) hit rate of occurrence records under different models after CDF normalization.

To reduce the influence of differences in output distributions between MCA and SDMs, all continuous suitability outputs were further standardized to a common probability scale using CDF normalization before comparison. After normalization, clear differences remained in the area structure of the four suitability classes among models (Figure 5a). The three MCA models showed highly consistent area proportions, with unsuitable, slightly suitable, moderately suitable, and highly suitable habitats accounting for 24.6%, 35.4%, 20.0%, and 20.0%, respectively. In contrast, variation among SDMs was mainly reflected in the proportions of unsuitable and slightly suitable habitats. MaxEnt showed the strongest concentration toward unsuitable habitats, with 49.74% classified as unsuitable and only 9.61% as slightly suitable, whereas moderately suitable and highly suitable habitats accounted for 20.6% and 20.0%, respectively. XGBoost produced a more balanced structure, with 35.44% unsuitable, 21.19% slightly suitable, 22.35% moderately suitable, and 21.02% highly suitable habitats. The area structures of GLM and the ensemble model were more similar to those of the MCA models.

The distribution of occurrence records among suitability classes also differed between the two modeling approaches (Figure 5b). AHP, TOPSIS, and OWA (α = 2) produced highly similar results, with more than 95% of occurrence records located in the moderately and highly suitable habitats, only 3.6% to 5.0% in slightly suitable habitats, and less than 0.15% in unsuitable habitats. In comparison, the SDMs generally showed higher hit proportions in highly suitable habitats. The proportions of occurrence records falling in highly suitable habitats were 89.67% for GLM, 90.54% for MaxEnt, 91.67% for XGBoost, and 90.27% for the ensemble model, whereas the corresponding proportions in moderately suitable habitats were 9.93%, 7.86%, 6.99%, and 9.46%, respectively. In all four SDMs, the combined hit proportion in moderately and highly suitable habitats exceeded 98%, while the proportions in unsuitable and slightly suitable habitats remained very low. In GLM, no occurrence record fell within unsuitable habitats, and only 0.4% occurred in slightly suitable habitats. For MaxEnt, XGBoost, and the ensemble model, the proportions of occurrence records in unsuitable and slightly suitable habitats were all below 1%.

**Figure 6:**
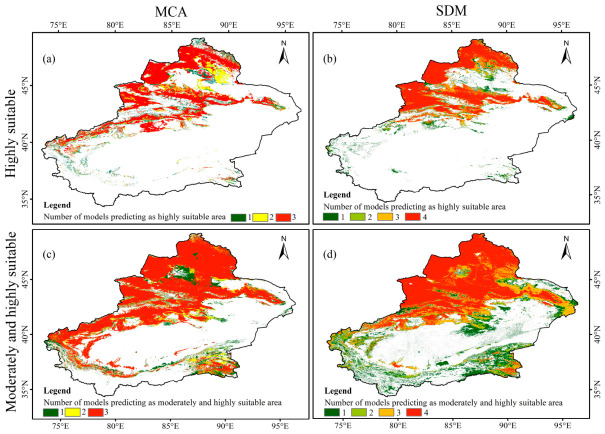
Spatial agreement of suitable habitats identified by multi-criteria analysis (MCA) and species distribution models (SDMs): (**a**) highly suitable habitats identified by MCA; (**b**) highly suitable habitats identified by SDMs; (**c**) combined moderately and highly suitable habitats identified by MCA; (**d**) combined moderately and highly suitable habitats identified by SDMs.

**Figure 7:**
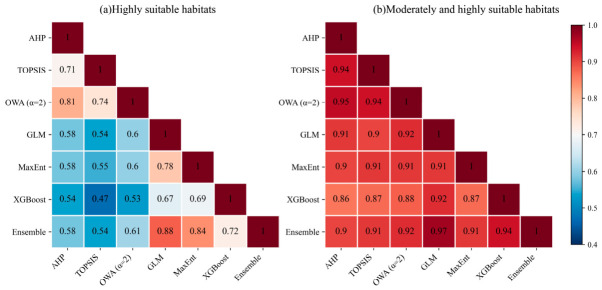
Pairwise Jaccard indices of spatial overlap among different models for (**a**) highly suitable habitats and (**b**) combined moderately and highly suitable habitats.

To examine the spatial consistency of locust suitable habitat identification among different models, spatial agreement maps were constructed for highly suitable habitats and for the combined moderately and highly suitable habitats within MCA and SDMs, respectively (Figure 6), and the spatial overlap among models was further quantified using the Jaccard index (Figure 7). At the highly suitable habitat level, both MCA and SDMs formed obvious high-consistency patches on the northern slopes of the Tianshan Mountains, in the Ili River Valley, and along the margins of the Junggar Basin, but their spatial coverage and connectivity differed (Figure 6a,b). High-consistency areas from MCA were more continuous and belt-like, whereas those from SDMs were more concentrated in river valley oases, piedmont plains, and surrounding transition zones with dense historical locust occurrences. Jaccard indices further quantified this difference (Figure 7a). Within MCA, pairwise agreement was relatively high, with values of 0.81 for AHP versus OWA and 0.74 for TOPSIS versus OWA. Within SDMs, agreement was also strong, with values of 0.88 for GLM versus the ensemble model and 0.84 for MaxEnt versus the ensemble model. In contrast, the Jaccard indices between MCA and SDMs were substantially lower, mostly ranging from 0.47 to 0.61, indicating clear differences in the fine spatial delineation of highly suitable habitats.

When the suitability range was expanded to include both moderately and highly suitable habitats, spatial agreement increased markedly among all models (Figure 6c,d and Figure 7b). Pairwise Jaccard indices within MCA and within SDMs were generally above 0.90, and those between MCA and SDMs remained stable at 0.88 to 0.92, corresponding to the large continuous high-consistency areas shown in Figure 6c,d. These results indicate that MCA and SDMs were broadly consistent in the macro-scale identification of potential suitable habitats, whereas systematic differences remained in the fine spatial positioning of highly suitable habitats.

## 4. Discussion

### 4.1. Differences in Predictive Performance Between MCA and SDMs

Although SDMs produced higher AUC and TSS values than MCA in this study, this result should not be interpreted as evidence that SDMs are generally superior to MCA. SDMs are occurrence-based models that are trained to distinguish occurrence records from pseudo-absence samples. Therefore, their outputs are more directly aligned with point-based discrimination metrics such as AUC and TSS. In contrast, MCA is a knowledge-driven suitability assessment framework that integrates ecological knowledge, environmental suitability, and decision preferences. Its purpose is to identify potential suitable habitats rather than to maximize point-based classification accuracy. The lower AUC and TSS values of MCA thus mainly reflect differences in modeling logic and evaluation framework, rather than lower overall applicability.

AUC and TSS should be interpreted as internal point-based discrimination indicators, not as complete measures of ecological validity, transferability, or management usefulness. Similar findings have been reported in comparisons between expert-based and correlative models, where correlative models often show stronger discrimination when occurrence data are sufficient, while expert-based approaches remain useful under limited data conditions [10,56]. Model performance metrics should be interpreted together with the intended application context rather than used as the only basis for model selection.

### 4.2. Spatial Consistencies and Differences Between MCA and SDMs in Identifying Locust Suitable Habitats

MCA and SDMs showed broad spatial agreement in identifying locust-suitable habitats in Xinjiang, China. Both approaches consistently identified the southern slopes of the Altai Mountains, the central northern slopes of the Tianshan Mountains, the Ili River Valley, and the margins of the Junggar Basin as major suitable habitat regions. This indicates that the macro-scale pattern of locust suitability is largely constrained by shared environmental gradients, including thermal conditions, moisture availability, vegetation background, and terrain. Together, these conditions define a broad environmental niche space for dominant grassland locusts in Northern Xinjiang, China. Similar spatial patterns have also been reported in previous studies on typical locust species in Xinjiang, China and adjacent regions [24,25,26,27].

However, clear differences were observed in the delineation of highly suitable habitats. After CDF normalization, MCA and SDMs were highly consistent for the combined moderately and highly suitable habitats, whereas their overlap was lower for highly suitable habitats alone. MCA tended to produce broader and more continuous suitability zones, while SDMs identified more concentrated hotspots in piedmont areas, river valleys, and transition zones with dense historical occurrence records. This reflects the difference between expert-defined potential suitability and occurrence-constrained realized patterns. MCA can represent environmentally suitable areas even when occurrence records are sparse, whereas hotspots identified by SDMs may reflect environmental suitability as well as dispersal limitation, local habitat heterogeneity, population persistence, historical monitoring intensity, and management activities. Therefore, the differences between MCA and SDMs should be interpreted as different dimensions of locust habitat suitability rather than only as technical inconsistencies.

### 4.3. Applicability of MCA and SDMs to Locust Monitoring and Management

The differences between MCA and SDMs have practical implications for locust monitoring and management. Model choice should depend on the management objective and data availability. MCA is more suitable for rapid regional screening and environmental suitability assessment when occurrence records are limited or unevenly distributed. SDMs are more suitable for fine-scale hotspot refinement when sufficient and reliable occurrence records are available. These results are consistent with broader studies showing that locust management requires spatially explicit information for early warning, surveillance, and timely control actions [3,4,5].

For practical decision-making, MCA can first be used to delineate broad priority zones for monitoring, and SDMs can then refine core suitable habitats within these zones. Areas identified as moderately or highly suitable by both approaches should be prioritized for field surveys and control planning. Areas identified only by MCA may require field verification, while areas identified as highly suitable by SDMs can be treated as priority sites for intensive monitoring and targeted control. This complementarity provides a basis for future hybrid frameworks that combine broad environmental suitability screening with occurrence-based hotspot refinement.

### 4.4. Limitations and Uncertainties

Although this study provides a comparative assessment of MCA and SDMs for identifying locust suitable habitats in Xinjiang, China, several limitations and uncertainties should be noted. The occurrence records of multiple dominant locust species were combined to represent regional locust suitability. This treatment was consistent with the objective of integrated habitat assessment at the regional scale, but it may obscure species-specific ecological responses and niche differences. Different species may respond differently to moisture, vegetation type, soil texture, overwintering environment, and microhabitat conditions. Because the combined dataset was dominated by *Calliptamus italicus* and *Gomphocerus sibiricus*, the composite suitability pattern may underrepresent *Locusta migratoria manilensis*. Therefore, the results should be interpreted as a composite suitability pattern for dominant grassland locusts rather than as species-specific distribution maps. In addition, because the environmental variables mainly represented multi-year average conditions, the predicted suitable habitats should be interpreted as long-term potential suitability patterns, not as outbreak risk maps for specific years. This temporal aggregation may smooth short-term weather anomalies, vegetation changes, and annual variation in locust population processes. Future studies should use temporally matched occurrence records and seasonal environmental variables to assess annual outbreak risk.

Uncertainty also remains in the MCA and SDM frameworks. For MCA, the suitability grading criteria and AHP weights were based on published studies, locust ecological preferences, and regional environmental knowledge, but some subjectivity is unavoidable. Different weighting schemes or grading criteria may influence the spatial extent and class structure of predicted suitable habitats. The OWA scenarios with different α values indicated that MCA outputs varied under different decision attitudes, although they did not replace a formal sensitivity analysis of AHP weights. For SDMs, occurrence records may still contain sampling bias, because field surveys and published records are often concentrated in accessible areas or historically monitored regions. Such uneven sampling may cause models to overrepresent well-surveyed habitats and underrepresent suitable but poorly sampled areas. Pseudo-absence points do not represent confirmed absences. Although the same pseudo-absence set was used for all SDMs to improve comparability, results may still be affected by random sampling, sampling extent, the presence-to-pseudo-absence ratio, and algorithm settings. If pseudo-absence points are sampled from environmentally suitable but unsurveyed areas, model discrimination metrics such as AUC and TSS may be affected, and the spatial boundary of predicted suitable habitats may shift. Therefore, the SDM results should be interpreted as occurrence-constrained suitability patterns rather than definitive absence-presence classifications.

## 5. Conclusions

This study compared multi-criteria analysis (MCA) and species distribution models (SDMs) for identifying locust suitable habitats in Xinjiang, China, using a unified environmental variable system and occurrence dataset. By integrating climate, vegetation, soil, hydrological, and topographic variables, the study evaluated differences between the two approaches in predictive performance, spatial pattern identification, and practical applicability for locust monitoring and management.

The results showed that both MCA and SDMs effectively identified the main spatial pattern of locust suitable habitats in Xinjiang, China. The southern slopes of the Altai Mountains, the central northern slopes of the Tianshan Mountains, the Ili River Valley, and the margins of the Junggar Basin were consistently recognized as the major suitable habitat regions. Although SDMs generally produced higher AUC and TSS values, this difference should not be interpreted as the absolute superiority of one approach over the other, because MCA and SDMs are based on different modeling logics and reflect different aspects of habitat suitability. After CDF normalization, the two approaches showed high consistency in the identification of broad suitable habitats but differed clearly in the delineation of highly suitable habitats. In general, MCA tended to identify broader and more continuous suitable habitat belts, whereas SDMs produced more concentrated highly suitable habitats.

Overall, this study indicates that MCA and SDMs are complementary rather than substitutive approaches in locust habitat identification. MCA is more suitable for broad-scale screening when occurrence records are limited, whereas SDMs are more suitable for refining core suitable habitats when reliable occurrence data are available. The methodological contribution of this study is that it compares knowledge-driven MCA and occurrence-driven SDMs under a unified framework and shows that the two approaches provide different but complementary information. MCA helps identify broad potential environmental suitability, while SDMs refine occurrence-constrained hotspots. Future studies could develop hybrid frameworks that integrate expert-based suitability rules with occurrence-driven model calibration to improve locust habitat mapping and risk-based management.

## Figures and Tables

**Table 1:** Data sources.

Category	Variables	Temporal Resolution	Spatial Resolution	Source
Climate	MOD11A2.061 LST	8 days	1 km	Google Earth Engine (https://developers.google.com/earth-engine/datasets, accessed on 15 November 2025)
	Air temperature and Precipitation	Monthly	1 km	National Tibetan Plateau Data Center (https://data.tpdc.ac.cn/, accessed on 15 November 2025)
	Soil moisture	Monthly	0.04°	TerraClimate (https://www.climatologylab.org/terraclimate.html, accessed on 15 November 2025)
Vegetation	MOD13A3.061 NDVI	Monthly	1 km	Google Earth Engine (https://developers.google.com/earth-engine/datasets, accessed on 15 November 2025)
	Land cover type	—	Vector	GLC2000 (https://forobs.jrc.ec.europa.eu/, accessed on 15 November 2025)
Soil	Clay content	—	250 m	SoilGrids V2.0 (https://soilgrids.org, accessed on 15 November 2025)
	Sand content			
	pH			
Hydrological	Rivers and lakes	—	Vector	HydroSHEDS (https://www.hydrosheds.org, accessed on 15 November 2025)
Topographic	SRTM DEM	—	90 m	Geospatial Data Cloud (http://www.gscloud.cn, accessed on 15 November 2025)

**Table 2:** Suitability grading criteria for environmental variables used in the MCA framework.

Variables	Score 1	Score 2	Score 3	Score 4	Score 5
Development period temperature (°C)	0–4, >30	4–8, 28–30	8–12, 25–28	12–16, 22–25	16–22
Development period precipitation (mm)	0–100, ≥800	100–160, 650–800	160–220, 520–650	220–280, 420–520	280–420
Overwintering period LST (°C)	<−20	−20 to −10	−10 to −5	−5 to 0	≥0
Overwintering period precipitation (mm)	0–50, ≥200	150–200	50–90	120–150	90–120
Development period NDVI	0–0.10, ≥0.65	—	0.10–0.15, 0.55–0.65	0.15–0.25, 0.45–0.55	0.25–0.45
Land cover type	Other	Desert, forest	Gravels	Farmland	Grassland, meadow
Aspect	North	Northeast, northwest	Flat	East, west	South, southwest, southeast
Slope (°)	>15	12–15	8–12	4–8	0–4
Clay content (g/kg)	0–80, ≥480	80–120, 400–480	120–160, 320–400	160–200, 260–320	200–260
Sand content (g/kg)	0–100, ≥650	100–160, 550–650	160–220, 450–550	220–280, 360–450	280–360
Distance to rivers (km)	>2.5	2.0–2.5	1.0–2.0	0.4–1.0	0–0.4
Distance to lakes (km)	>3.0	2.2–3.0	1.4–2.2	0.8–1.4	0–0.8

**Table 3:** Evaluation metrics for different models.

Model	AUC	TSS
AHP	0.937	0.707
TOPSIS	0.936	0.697
OWA (α = 0.0001)	0.900	0.652
OWA (α = 0.1)	0.906	0.659
OWA (α = 0.75)	0.931	0.693
OWA (α = 1)	0.937	0.707
OWA (α = 1.25)	0.940	0.722
OWA (α = 2)	0.943	0.720
OWA (α = 5)	0.939	0.726
OWA (α = 10)	0.932	0.721
OWA (α = 10,000)	0.632	0.263
GLM	0.979	0.836
MaxEnt	0.974	0.841
XGBoost	0.978	0.851
Ensemble	0.982	0.848

## Data Availability

The data and materials underlying this article are available in the article.

## Appendix A

**Table A1:** Hierarchical structure and attribute weights based on AHP.

Goal Layer	Criterion Layer	Criterion Weight	CR	Indicator Layer	Indicator Weight	CR	Final Weight
Locust habitat suitability	Climate	0.4935	0.0419	Development period temperature	0.3889	0.0164	0.1919
				Development period precipitation	0.1535		0.1808
				Overwintering period LST	0.0687		0.0757
				Overwintering period precipitation	0.3839		0.0339
	Vegetation	0.2432		Development period NDVI	0.6667	0	0.1621
				Land cover type	0.3333		0.0811
	Soil	0.1222		Clay content	0.3333	0	0.0407
				Sand content	0.6667		0.0815
	Topography	0.0466		Slope	0.75	0	0.0350
				Aspect	0.25		0.0117
	Hydrology	0.0944		Distance to rivers	0.5	0	0.0472
				Distance to lakes	0.5		0.0472

**Table A2:** Proportion of suitable habitat areas based on MCA.

Model	Area Proportion (%)			
	Unsuitable	Slightly Suitable	Moderately Suitable	Highly Suitable
AHP	35.44	46.49	14.88	3.18
TOPSIS	44.8	41.2	12.41	1.59
OWA (α = 0.0001)	0	1.29	0	98.71
OWA (α = 0.1)	0.08	1.21	1.24	97.47
OWA (α = 0.75)	21.72	50.71	22.83	4.75
OWA (α = 1)	35.44	46.49	14.88	3.18
OWA (α = 1.25)	40.28	46.85	10.69	2.18
OWA (α = 2)	45.79	47.82	5.69	0.7
OWA (α = 5)	58.24	39.94	1.76	0.06
OWA (α = 10)	70.51	28.82	0.66	0.02
OWA (α = 10,000)	92.27	6.77	0.89	0.07

**Table A3:** Proportion of suitable habitat areas based on SDMs.

Model	Area Proportion (%)			
	Unsuitable	Slightly Suitable	Moderately Suitable	Highly Suitable
GLM	65.98	12.83	6.17	15.02
MaxEnt	82.26	8.44	2.01	7.29
XGBoost	62.35	18.25	3.22	16.18
Ensemble	66.71	19.4	4.25	9.64
